# Exogenous Melatonin Confers Salt Stress Tolerance to Watermelon by Improving Photosynthesis and Redox Homeostasis

**DOI:** 10.3389/fpls.2017.00295

**Published:** 2017-03-01

**Authors:** Hao Li, Jingjing Chang, Hejie Chen, Zhongyuan Wang, Xiurong Gu, Chunhua Wei, Yong Zhang, Jianxiang Ma, Jianqiang Yang, Xian Zhang

**Affiliations:** College of Horticulture, Northwest A&F UniversityYangling, China

**Keywords:** melatonin, photosynthesis, redox homeostasis, salt stress, watermelon

## Abstract

Melatonin, a pleiotropic signal molecule, has been shown to play important roles in the regulation of plant growth, development, and responses to environmental stresses. Since a few species have been investigated to unveil the effect of exogenous melatonin on salt stress, the underlying mechanism of melatonin-mediated salt stress tolerance in other plant species still remains largely unknown. In this study, the effects of melatonin on leaf photosynthesis and redox homeostasis in watermelon were examined under salt stress (300 mM NaCl) along with different doses of melatonin (50, 150, and 500 μM) pretreatment. NaCl stress inhibited photosynthesis and increased accumulation of reactive oxygen species and membrane damage in leaves of watermelon seedlings. However, pretreatment with melatonin on roots alleviated NaCl-induced decrease in photosynthetic rate and oxidative stress in a dose-dependent manner. The protection of photosynthesis by melatonin was closely associated with the inhibition of stomatal closure and improved light energy absorption and electron transport in photosystem II, while the reduction of oxidative stress by melatonin was attributed to the improved redox homeostasis coupled with the enhanced activities of antioxidant enzymes. This study unraveled crucial role of melatonin in salt stress mitigation and thus can be implicated in the management of salinity in watermelon cultivation.

## Introduction

Since plants cannot relocate, they have to endure multiple biotic and abiotic stresses throughout their life cycle. Among these stresses, soil salinity is one of the most important environmental hazards that inhibit plant growth and development, causing significant yield losses, particularly in arid and semi-arid areas (Evelin et al., 2009; Porcel et al., 2012). Salinity adversely affects plant physiology through multiple mechanisms. Firstly, increased accumulation of sodium ions (Na^+^) cause damage to cellular organelles, inhibit protein synthesis and enzyme activities, and uncouple photosynthesis and respiration; secondly, salinity decreases nutrient uptake and/or transport to the shoot, resulting in a nutrient imbalance; and thirdly, salinity decreases soil osmotic potentials and hinders water uptake by roots, leading to a physiological drought in the plant (Ruiz-Lozano et al., 2012).

Photosynthesis, the most important physico-chemical process accountable for the energy production in higher plants, is very sensitive to salt stress (Meloni et al., 2003). During salt stress, the intercellular CO_2_ concentration in leaf is decreased due to stomatal closure. In addition, salt stress reduces consumption of NADPH by the Calvin cycle, inhibits chlorophyll synthesis and Rubisco activity, and disrupts the photosynthetic electron transport. Notably, salinity-induced inhibition of the photosynthetic electron transport results in excessive accumulation of toxic reactive oxygen species (ROS) such as O_2_^•-^, H_2_O_2_, and ●OH and disruption of cellular redox homeostasis. Over accumulation of ROS promotes degradation of chlorophyll and reduces photochemical efficiency of photosystem II (PSII) forming a vicious cycle (Woo et al., 2004; Allakhverdiev et al., 2008). Moreover, as strong oxidant, ROS at high concentration can damage membranes through lipid peroxidation, break DNA strand, and inactivate various vital enzymes (Cheng and Song, 2006).

Melatonin (*N*-acetyl-5-methoxytryptamine), a pleiotropic and highly conserved molecule, is ubiquitous throughout the animal and plant kingdoms (Hardeland et al., 2011). Since the discovery of melatonin in vascular plants in 1995 (Dubbels et al., 1995; Hattori et al., 1995), numerous subsequent studies have demonstrated its important roles in regulating plant growth, development, and defense against various environmental stresses (Arnao and Hernández-Ruiz, 2014, 2015; Zhang et al., 2015; Nawaz et al., 2016). The beneficial role of melatonin in stress mitigation is broadly attributable to higher photosynthesis, improvement of cellular redox homeostasis and alleviation of oxidative stress, and regulation of the expression of stress-responsive genes involved in signal transduction (Li et al., 2012; Bajwa et al., 2014; Zhang and Zhang, 2014). Recently, several studies have shown that salt stress can increase melatonin content in roots (Arnao and Hernández-Ruiz, 2009; Mukherjee et al., 2014) and exogenous application of melatonin enhances salt stress tolerance in *Malus hupehensis* and *Helianthus annuus* (Li et al., 2012; Mukherjee et al., 2014). However, it is still unclear whether such response of melatonin against salt stress is universal for other plant species. Furthermore, the mechanism of melatonin-mediated salt stress tolerance is obscure.

Watermelon (*Citrullus lanatus* L.), one of the economically important crops in the world, is a high water-consuming plant and is very sensitive to salt stress (Yetişir and Uygur, 2009). In the present study, we tried to understand the regulatory mechanism controlling melatonin-mediated salt stress tolerance in watermelon in terms of photosynthesis and redox homeostasis. The response of photosynthetic process including stomatal movement, energy absorption, photosynthetic electron transport, and CO_2_ assimilation and the response of redox homeostasis including ROS-generation and -scavenging under salt stress were investigated. This study provides a novel physiological basis for further dissection of the regulatory mechanism of melatonin-mediated salt stress tolerance in watermelon.

## Materials and Methods

### Plant Material and Treatments

Seeds of watermelon (*Citrullus lanatus* L. cv. 04-1-2) were provided by the Watermelon and Melon Research Group at Northwest A&F University, Yangling, China. Seeds were surface sterilized with 5% sodium hypochlorite for 5 min, pre-soaked at 25°C sterile water for 10 h and then placed on moist filter paper in Petri dish in the dark at 30°C for germination. Germinated seeds were sown in plastic pots (10 cm × 7 cm × 8.5 cm) filled with a mixture of 2:1 (v/v) autoclaved sand and commercial peat-based compost (Shaanxi Yufeng Seed Industry Co., Ltd., Yangling, China). The seedlings were pre-cultured under natural light in a greenhouse at Northwest A&F University, Yangling, China (34°28′N, 108°067′E) where the relative humidity was 65–95%, and the temperature was 28–35°C/16–20°C (day/night). All seedlings were uniformly watered daily and fertilized weekly with 1/2 strength Hoagland’s solution.

Seedlings at the three-leaf stage were treated with 0, 50, 150, or 500 μM melatonin (80 mL per plant) on roots for 6 days (once every 2 days). The melatonin (Sigma–Aldrich, St. Louis, MO, USA) solutions were prepared by dissolving the solute in ethanol followed by dilution with Milli-Q water [ethanol/water (v/v) = 1/10000]. After melatonin pretreatment, plants were irrigated with 300 mM NaCl (80 mL per plant). Seven days later, samples of leaf (the second fully expanded leaf beneath the growing point) were harvested after measuring the gas exchange and chlorophyll fluorescence parameters. Harvested samples were rapidly frozen in liquid nitrogen and stored at -80°C until the biochemical assay.

### Gas Exchange and Chlorophyll Content Measurements

The gas exchange of attached leaves was measured using an infrared gas analyzer, Li-Cor-6400 (Li-Cor Inc., Lincoln, NE, USA) equipped with an LED red/blue light source (6400-02B). The photosynthetic photon flux density (PPFD) was set at 1000 μmol m^-2^ s^-1^ and the cuvette air flow rate was 500 ml min^-1^. Net photosynthetic rate (Pn) and stomatal conductance (Gs) were recorded simultaneously.

Photosynthetic pigments from leaf were extracted in 80% acetone and the contents of chlorophyll a and chlorophyll b were determined according to the method of Lichtenthaler and Wellburn (1983).

### Chlorophyll Fluorescence Measurements

Chlorophyll fluorescence parameters were measured in leaves with a Portable Chlorophyll Fluorometer (PAM2500; Heinz Walz, Effeltrich, Germany) after a 30 min dark-adaptation. The maximum photochemical efficiency of PSII (*F*v/*F*m), actual photochemical efficiency of PSII [Y(II)], photochemical quenching (qP), and non-photochemical quenching [Y(NPQ)] were calculated according to Pfündel et al. (2008).

### Analysis of H_2_O_2_, O_2_^•-^, Malondialdehyde, and Relative Electrolyte Leakage

H_2_O_2_ content was measured according to the method of Willekens et al. (1997) with a slight modification. Briefly, 0.3 g of leaf samples was homogenized with 3 mL of 1 M HClO_4_ at 4°C, and the homogenate was centrifuged at 6,000 × *g* for 5 min at 4°C. pH of the supernatant was adjusted to 6.0–7.0 with 4 M KOH and centrifuged at 12,000 × *g* for 5 min at 4°C. Afterward, the supernatant was passed through an AG1x8 pre-packed column (Bio-Rad, Hercules, CA, USA) and H_2_O_2_ was eluted with 4 mL double-distilled H_2_O. The sample (800 μL) was mixed with 400 μL reaction buffer containing 4 mM 2,2′-azino-di (3-ethylbenzthiazoline-6-sulfonic acid) and 100 mM potassium acetate at pH 4.4, 400 μL deionized water and 0.25 U of horseradish peroxidase (HRP). H_2_O_2_ content was measured at OD_412_.

Superoxide production was quantified according to the method of Elstner and Heupel (1976) with a slight modification. 0.5 g of leaf sample was homogenized with 3 mL of 65 mM potassium phosphate buffer (pH 7.8). After centrifugation, 1 mL of the supernatant was mixed with 0.9 mL of 65 mM phosphate buffer (pH 7.8) and 0.1 mL of 10 mM hydroxylamine hydrochloride. After incubation at 25°C for 20 min, 17 mM sulfanilamide and 7 mM α-naphthylamine were added to the incubation mixture and reaction at 25°C for 20 min. Then, ethyl ether in the same volume was added and centrifuged at 1,500 × *g* for 5 min. The absorbance was recorded at 530 nm. Sodium nitrite was used as a standard solution to calculate the production rate of superoxide.

Malondialdehyde (MDA) as an end product of lipid peroxidation was measured according to the method of Hodges et al. (1999). Leaf sample (0.3 g) was homogenized in 5 mL of 10% (w/v) trichloroacetic acid (TCA) and the homogenate was centrifuged at 3,000 × *g* for 10 min. Then, 4 mL of 20% TCA containing 0.65% (w/v) TBA was added to 1 mL of supernatant. The mixture was heated at 95°C for 25 min and immediately cooled to stop the reaction. After centrifugation at 3,000 × *g* for 10 min, the absorbance of the supernatant was recorded at 440, 532, and 600 nm.

The relative electric leakage (REL) was measured and calculated as described previously by Zhou and Leul (1998). The second fully expanded leaf beneath the growing point (1.0 g) were cut into 0.5-cm circles using a punch and placed in a 50-mL test tube containing 25-mL deionized water. Afterward, the test tubes were vacuumed for 10 min, the leaf samples were immersed and vibrated 20 min, and then measured the conductivity of the solution (C_1_) using a conductivity meter (DDS-2307). Then samples were boiled for 10 min and the conductivity (C_2_) was measured again when the solution was cooled to room temperature. REL was calculated as C_1_/C_2_ × 100%.

### AsA and GSH Determination

Reduced glutathione (GSH) and oxidized glutathione (GSSG) contents were determined according to Rao et al. (1995) by an enzymatic recycling method. Leaf sample (0.3 g) was homogenized in 2 mL of 5% metaphosphoric acid containing 2 mM EDTA and centrifuged at 4°C for 15 min at 12,000 × *g*. For the total glutathione assay, 0.1 mL of the supernatant was added to a reaction mixture containing 0.2 mM NADPH, 100 mM phosphate buffer (pH 7.5), 5 mM EDTA, 0.6 mM 5,5′-dithio-bis (2-nitrobenzoic acid). The reaction was started by adding 3 U of GR and was monitored by measuring the changes in absorbance at 412 nm for 1 min. For the GSSG assay, GSH was masked by adding 20 μL of 2-vinylpyridine for 1 h at 25°C. The GSH concentration was obtained by subtracting the GSSG concentration from the total concentration.

Ascorbic acid (AsA) and dehydroascorbic acid (DHA) were measured following the method of Law et al. (1983). Leaf sample (0.3 g) was homogenized in cold 6% (w/v) TCA. For total ascorbate (AsA+DHA) content assay, the extract was incubated with 150 mM phosphate buffer solution (pH 7.4) and 10 mM DTT for 20 min to reduce all DHA to AsA and then 100 μL of 0.5% (w/v) *N*-ethylmaleimide (NEM) was added to remove excess DTT. For AsA content assay, 200 μL deionized H_2_O was substituted for DTT and NEM. Then the reaction mixtures were added 400 μL 10% (w/v) TCA, 400 μL 44% phosphoric acid (v/v), 400 μL 70% (v/v) α′-dipyridyl in ethanol, and 200 μl 3% (w/v) FeCl_3_. The reaction mixtures were then incubated at 37°C for 60 min in a water bath and the absorbance was recorded at 525 nm. The DHA concentration was obtained by subtracting the AsA concentration from the total concentration.

### Antioxidant Enzyme Extraction and Activity Assays

Antioxidant enzyme activities were assayed in leaves by using spectrophotometric methods. For extraction of enzymes, frozen leaf sample (0.3 g) was ground with 3 mL ice-cold 25 mM HEPES buffer (pH 7.8) containing 0.2 mM EDTA, 2 mM AsA, and 2% PVP. The homogenates were centrifuged at 4°C for 20 min at 12,000 × *g*, and the resulting supernatants were used for the determination of enzymatic activity. Protein contents were determined following the method of Bradford and Williams (1976).

Superoxide dismutase (SOD) activity was assayed according to the method of Stewart and Bewley (1980) based on photochemical reduction of nitro blue tetrazolium (NBT). One unit of SOD activity was defined as the amount of enzyme required to cause 50% inhibition of the reduction rate of NBT as monitored at 560 nm. According to the procedure described by Cakmak and Marschner (1992), catalase (CAT) activity was measured in a reaction mixture containing 25 mM phosphate buffer (pH 7.0), 10 mM H_2_O_2_, and the enzyme extract. A decline in 240 nm was monitored. Ascorbate peroxidase (APX) and dehydroascorbate reductase (DHAR) activities were measured according to the method of Nakano and Asada (1981). The reaction mixture for APX contained 50 mM phosphate buffer (pH 7.0), 0.1 mM EDTA, 0.5 mM AsA, 1 mM H_2_O_2_, and enzyme extract. The reaction was initiated by adding H_2_O_2_ and the decrease in absorbance at 290 nm was monitored for 1 min. The reaction solution for DHAR contained 50 mM phosphate buffer, pH 7.0, 2.5 mM GSH, 0.1 mM EDTA, 0.2 mM dehydroascorbate (DHA), and enzyme extract. The reaction was initiated by adding DHA and the change in absorbance at 265 nm was monitored for 1 min. Monodehydroascorbate reductase (MDHAR) activity was assayed by monitoring the decrease in absorbance at 340 nm owing to reduced nicotinamide adenine dinucleotide (NADH) oxidation (Arrigoni et al., 1981). The assay solution contained 50 mM HEPES-KOH, pH 7.6, 2.5 mM AsA, 0.1 mM NADH, 0.5 U AsA oxidase, and 100 μL enzyme extract. The reaction was initiated by adding AsA oxidase.

### Statistical Analysis

The experiment was a completely randomized design with three replicates. Each replicate contained at least 10 plants. Analysis of variance (ANOVA) was used to test for significance, and significant differences (*P* < 0.05) between treatments were determined using Tukey’s test.

## Results

### The Effects of Melatonin on Photosynthesis in Watermelon under Salt Stress

**FIGURE 1:**
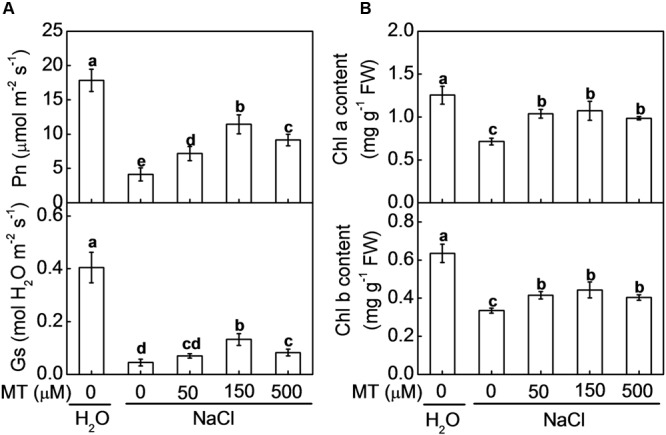
**Changes in (A)** the net photosynthetic rate (Pn) and stomatal conductance (Gs) and **(B)** contents of chlorophyll a and chlorophyll b in watermelon plants as influenced by salt stress alone or combined with melatonin pretreatment. All data were determined on the seventh day after NaCl treatment. The bars (means ± SD, *n* = 3) labeled with different letters are significantly different at *P* < 0.05 according to Tukey’s test. MT, melatonin.

As shown in **Figure 1A**, NaCl treatment resulted in a decreased Pn and Gs in watermelon. However, pretreatment with various concentrations (50–500 μM) of melatonin obviously alleviated salt stress-induced reduction in leaf Pn and Gs, whereas 150 μM melatonin appeared to be the most effective concentration in alleviating salt stress. For instance, after imposition of salt stress, Pn and Gs in the watermelon plants pretreated with 150 μM melatonin were reduced by 35.9 and 67.3%, respectively, which were fairly lower than those in the control plants, accounting for 76.7 and 88.9%, respectively. Both the higher and lower doses of melatonin were less effective in improving photosynthesis under salt stress. Similarly, chlorophyll a and chlorophyll b contents were decreased by NaCl stress, however, these decreases were alleviated by pretreatment with melatonin (**Figure 1B**).

**FIGURE 2:**
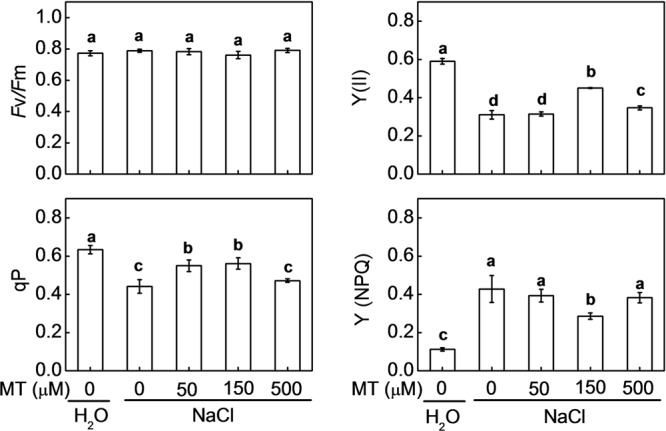
**Changes in chlorophyll fluorescence parameters in watermelon plants as influenced by salt stress alone or combined with melatonin pretreatment.** Data are the means ± SD of three replicates. Means denoted by the same letter did not differ significantly according to Tukey’s test (*P* < 0.05). MT, melatonin.

Leaf *F*v/*F*m remained almost unchanged under NaCl treatment alone or combined with melatonin pretreatment (**Figure 2**). Y(II) and qP were significantly decreased by NaCl stress. NaCl-induced decreases in Y(II) were alleviated by pretreatment with melatonin at 150 and 500 μM, more remarkably with the former one, while the decreases in qP were alleviated by pretreatment with melatonin at 50 and 150 μM, especially with the latter one. Y(II) and qP in the plants with 150 μM melatonin pretreatment were 45.4 and 27.2% higher than those in the control plants, after NaCl stress. However, Y(NPQ) which represents heat dissipation in PSII was induced by NaCl treatment, but this induction was attenuated by pretreatment with melatonin at 150 μM.

### The Effects of Melatonin on Oxidative Stress in Watermelon under Salt Stress

**FIGURE 3:**
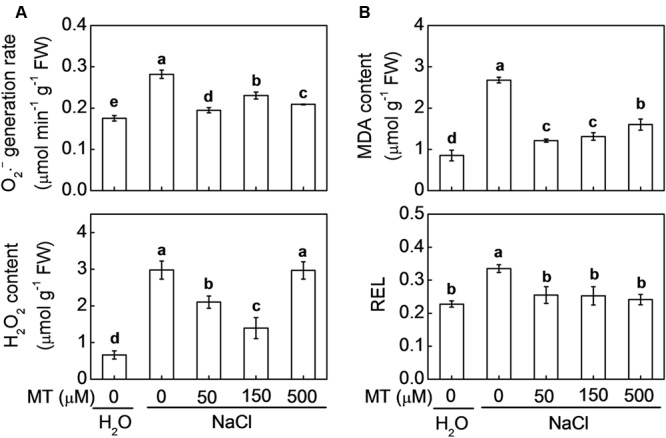
**(A)** Accumulation of O_2_^•-^ and H_2_O_2_ and **(B)** lipid peroxidation reflected by malondialdehyde (MDA) and relative electrolytic leakage (REL) in response to salt stress alone or combined with melatonin pretreatment. The bars (means ± SD, *n* = 3) labeled with different letters are significantly different at *P* < 0.05 according to Tukey’s test. MT, melatonin.

As shown in **Figure 3A**, O_2_^•-^ generation and H_2_O_2_ content were increased by NaCl stress. However, melatonin pretreatment significantly reduced NaCl-induced accumulation of O_2_^•-^ and H_2_O_2_. The optimum concentrations of melatonin for alleviating NaCl-induced accumulation of O_2_^•-^ and H_2_O_2_ were 50 and 150 μM, respectively. Similarly, both MDA and REL, which reflect damage to cell membrane, were significantly increased by NaCl stress in control plants, and these increases were attenuated by pretreatment with melatonin at 50, 150, or 500 μM (**Figure 3B**). For instance, mean values of O_2_^•-^, H_2_O_2_, MDA, and REL in the plants, pretreated with 150 μM melatonin followed by NaCl stress, were increased by 31.4, 110.4, 54.2, and 11.0%, respectively, far less than those in the control plants, accounting for 60.8, 348.9, 214.6, and 47.2%, respectively, after NaCl stress alone.

### The Effects of Melatonin on Antioxidant System in Watermelon under Salt Stress

**FIGURE 4:**
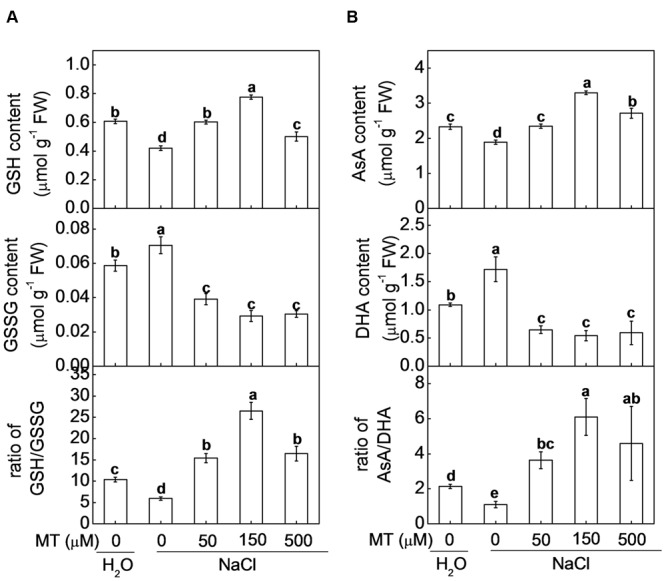
**(A)** Glutathione and **(B)** ascorbate homeostasis in watermelon plants as influenced by salt stress alone or combined with melatonin pretreatment. Data are the means ± SD of three replicates. Means denoted by the same letter did not differ significantly according to Tukey’s test (*P* < 0.05). MT, melatonin; GSH, reduced glutathione; GSSG, oxidized glutathione; AsA, ascorbic acid; DHA, dehydroascorbic acid.

Through evolution, plants have developed a set of antioxidant systems to remove excessive ROS that are harmful to plant cells. In control plants, the contents of GSH and AsA were decreased, but the contents of GSSG and dehydroascorbate (DHA) were increased by NaCl stress (**Figure 4**). As a result, the ratios of GSH/GSSG and AsA/DHA in control plants were dramatically reduced by NaCl stress. However, pretreatment with melatonin increased GSH and AsA contents but decreased GSSG and DHA contents under salt stress. Eventually, the ratio of GSH/GSSG and AsA/DHA were significantly higher in the plants pretreated with melatonin than those in control plants after NaCl stress. Moreover, the highest ratios of GSH/GSSG (26.5) and AsA/DHA (6.1) were found in the plants with 150 μM melatonin pretreatment after NaCl stress.

**FIGURE 5:**
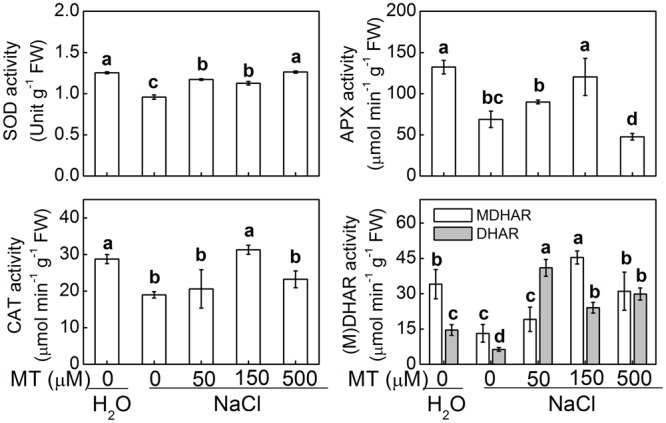
**The activities of antioxidant enzymes in response to salt stress alone or combined with melatonin pretreatment.** The bars (means ± SD, *n* = 3) labeled with different letters are significantly different at *P* < 0.05 according to Tukey’s test. MT, melatonin; SOD, superoxide dismutase; CAT, catalase; APX, ascorbate peroxidase; DHAR, dehydroascorbate reductase; MDHAR, monodehydroascorbate reductase.

The activities of major antioxidant enzymes such as SOD, CAT, APX, DHAR, and MDHAR were significantly decreased by NaCl stress in control plants (**Figure 5**). However, NaCl-induced decreases in CAT and APX were only alleviated by pretreatment with 150 μM melatonin, the decreases in SOD and DHAR were alleviated by pretreatment with 50, 150, or 500 μM melatonin, and the decrease in MDHAR was alleviated by pretreatment with 150 or 500 μM melatonin. Moreover, after NaCl treatment, the highest activities of CAT, APX, and MDHAR were recorded in the plants with 150 μM melatonin pretreatment, while the highest activities of SOD and DHAR were recorded in the plants with 500 and 50 μM melatonin pretreatment, respectively.

## Discussion

In recent years, melatonin has emerged as a research focus in plant science. Previous studies have shown that exogenous melatonin enhances salt stress tolerance in some plant species including *M. hupehensis* and *Glycine max* (Li et al., 2012; Mukherjee et al., 2014). Nevertheless, our knowledge regarding the mechanisms involved in melatonin-mediated tolerance to salt stress still remains fragmentary. In this study, we examined the effects of melatonin on photosynthesis and redox state in watermelon under NaCl stress. Our results indicate that application of melatonin on roots enhances watermelon tolerance to salt stress by improving photosynthesis and cellular redox homeostasis in leaves.

### Exogenous Melatonin Alleviated NaCl-Induced Inhibition in Photosynthesis

Photosynthesis is one of the key physiological processes highly sensitive to salt stress due to its detrimental effect on bioenergetic processes of photosynthesis (Takahashi and Murata, 2008). In the current study, NaCl stress inhibited photosynthesis and biomass accumulation in watermelon seedlings. However, in agreement with earlier study (Li et al., 2012), pretreatment with melatonin alleviated NaCl-induced inhibition in photosynthesis and biomass production and this role of melatonin was dose-dependent (**Figure 1** and **Supplementary Figure S1**). Melatonin with 500 or 50 μM concentration was less effective in improving photosynthesis and biomass accumulation under salt stress compared with the 150 μM concentration of melatonin. Under salt stress, one important response of plants is to close their stomata to minimize water loss, which is accompanied by notable decreases in Gs and consequently, stomatal limitation of photosynthesis (Brugnoli and Lauteri, 1991; Meloni et al., 2003). However, application of melatonin with optimal dose can improve the functions of stomata, by enabling plants to re-open their stomata under osmic stresses such as salt or drought stress (Li et al., 2014; Ye et al., 2016). Consistently, we also observed Gs was significantly decreased by NaCl stress, however, this decrease was alleviated by exogenous melatonin in a dose-dependent manner. Thus, alleviation of stomatal limitation by melatonin contributed to enhancement in photosynthesis under salt stress.

Photon flux is absorbed by the antenna pigments that excite chlorophyll. Part of the excitation energy is converted to redox energy via electron transport and leads to ultimate CO_2_ fixation, and the other is dissipated as heat and fluorescence emission (Strasser and Strasser, 1995). Decreases of chlorophyll contents and Y(II) and qP indicated that light energy absorption and electron transport in PS II were restricted by salt stress (**Figure 2**). Additionally, higher Y(NPQ) in salt-stressed plants also indicated that excitation energy was excessive for the capacity of electron transport. However, these decreases of chlorophyll contents, Y(II), and qP were alleviated by pretreatment with melatonin. Taken together, melatonin alleviated NaCl-induced inhibition in photosynthesis by regulating both stomatal and non-stomatal factors.

### Exogenous Melatonin Improved Cellular Redox Homeostasis under Salt Stress

Salt stress-induced decrease of PSII activity results in imbalance between the generation and utilization of electrons. To dissipate excessive light energy, excess electron is transported to molecular oxygen and thus generating ROS such as O_2_^•-^, ^1^O_2_, H_2_O_2_, and ●OH and other oxidants in the presence of respective reaction partners. Excess ROS can block the electron transport by inducing protein degradation and affecting the repair process of PSII, forming a vicious cycle (Allakhverdiev et al., 2008). Moreover, ROS could move to thylakoid and cell membranes, where they trigger levels of membrane lipid peroxidation and membrane permeability (Sharma et al., 2012). We found that salt stress-induced accumulation of O_2_^•-^ and H_2_O_2_ was consistent with the increase in MDA and REL, indicating that excess ROS might be responsible for the NaCl-induced membrane damage (**Figure 3**). Melatonin is a well-documented antioxidant and plays important roles in alleviating environmental stress-induced oxidative stress by scavenging most ROS or RNS (reactive nitrogen species) directly or indirectly in plants. In our previous study (Li et al., 2016), exogenous application of melatonin alleviated methyl viologen-induced photooxidative stress. In this study, we also found that salt stress-induced accumulation of ROS and ROS-caused membrane damage were alleviated by exogenous melatonin.

Strong evidence has demonstrated that melatonin is unable to directly scavenge O_2_^•-^ and H_2_O_2_ (Fowler et al., 2003; Bonnefont-Rousselot et al., 2011) and thus the regulation of redox homeostasis by melatonin results from its ability to induce antioxidant systems including antioxidant enzymes and non-enzymatic antioxidants. In plant cells, O_2_^•-^ is rapidly converted to H_2_O_2_ by the action of SOD, while H_2_O_2_ can be scavenged by an AsA and/or a GSH regenerating cycle and CAT (Noctor and Foyer, 1998). It is reported that exogenous melatonin increased AsA and GSH levels and redox status via upreglating activities of some key enzymes and alleviated dark- or methyl viologen-induced ROS accumulation and subsequent oxidative stress (Wang et al., 2012; Li et al., 2016). Consistently, we found GSH and AsA levels and redox status and the activities of some key enzymes (APX, DHAR, and MDAHR) involved in AsA-GSH cycle were significantly increased by melatonin at certain concentration (mostly by 150 uM) under salt stress (**Figures 4**, **5**). GSH is an essential co-substrate and reductant is required for regeneration of AsA (Foyer and Noctor, 2011) and cellular glutathione homeostasis has long been considered as a key element of signaling cascades, transducing information on environmental stress to their respective targets (May et al., 1998). In addition, Li et al. (2012) reported that melatonin-induced the activities of some antioxidant enzymes such as CAT under salt stress, which is well in agreement with our current study where melatonin promoted activities of SOD and CAT under salt stress (**Figure 5**). These results indicated that melatonin could improve cellular redox homeostasis by activating the entire antioxidant system in plants to protect cells from salt stress-induced oxidative stress.

**FIGURE 6:**
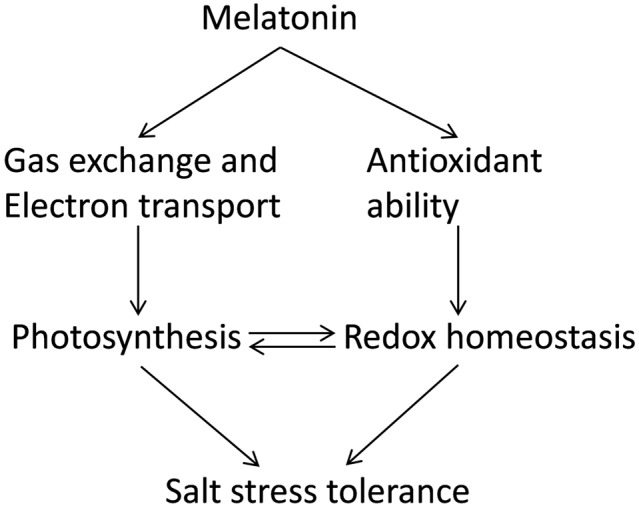
**A model showing potential mechanisms of melatonin-induced alleviation of NaCl-caused photosynthetic inhibition and oxidative stress in watermelon plants**.

To sum up, we have demonstrated that melatonin enhanced salt stress tolerance in watermelon seedlings in a dose-dependent manner. Under salt stress, melatonin increased photosynthesis by regulating stomatal movement and improving light energy absorption and electron transport in PSII. In addition, melatonin pretreatment improved redox homeostasis by inducing the activities of antioxidant enzymes and redox status of GSH/GSSG and AsA/DHA and subsequently reduced oxidative stress. Increased photosynthesis alleviated disruption of cellular redox homeostasis, while improved redox homeostasis contributed to keeping higher photosynthesis, forming a virtuous cycle (**Figure 6**).

## Author Contributions

HL and XZ designed research; HL, JC, HC, ZW, and XG performed research; HL, JC, XG, ZW, CW, JM, YZ, and JY analyzed data; HL, JC, and XZ wrote and revised the paper.

## Conflict of Interest Statement

The authors declare that the research was conducted in the absence of any commercial or financial relationships that could be construed as a potential conflict of interest.

## Supplementary Material

FIGURE S1 **Changes in biomass production in watermelon plants as influenced by salt stress alone or combined with melatonin pretreatment.** All data were determined on the seventh day after NaCl treatment. The bars (means ± SD, *n* = 5) labeled with different letters are significantly different at *P* < 0.05 according to Tukey’s test. MT, melatonin.
