# Supplementary figures and images for: Exogenous Melatonin Confers Salt Stress Tolerance to Watermelon by Improving Photosynthesis and Redox Homeostasis
Source: Front Plant Sci. 2017 Mar 1;8:295. doi: 10.3389/fpls.2017.00295 (PMC5331065; doi:10.3389/fpls.2017.00295)

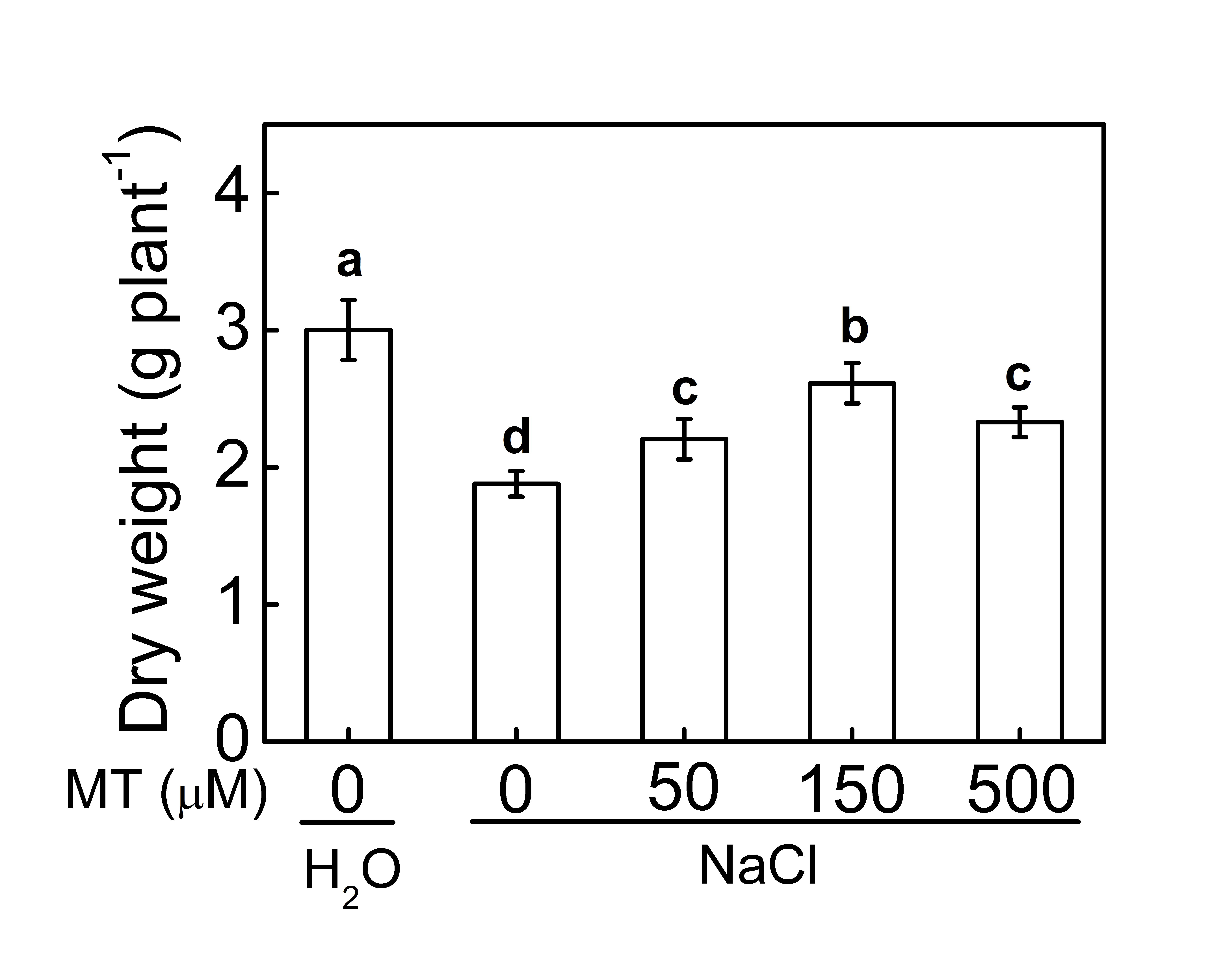

Supplement: FIGURE S1 — Changes in biomass production in watermelon plants as influenced by salt stress alone or combined with melatonin pretreatment. All data were determined on the seventh day after NaCl treatment. The bars (means ± SD, n = 5) labeled with different letters are significantly different at P < 0.05 according to Tukey’s test. MT, melatonin. [file Image_1.JPEG]
